# Explaining the Frequency Dependence of the DC-Biased Dielectric Response of Polar Nanoregions by Field-Enhanced Correlation Length

**DOI:** 10.3390/nano12081293

**Published:** 2022-04-11

**Authors:** Jianwei Zhang, Xiaoping Du, Jiguang Zhao, Yongsheng Duan

**Affiliations:** 1 Graduate Schools, Space Engineering University, Beijing 101416, China; quantum_pho@163.com; 2 Department of Electron & Optics Engineering, Space Engineering University, Beijing 101416, China; zhaoyy8600@163.com; 3 Department of Aerospace Science and Technology, Space Engineering University, Beijing 101416, China; duanys_vip@yeah.net

**Keywords:** dielectric, polar nanoregions, frequency dependence, correlation length

## Abstract

Understanding the effects of polar nanoregions (PNRs) dynamics on dielectric properties is a complex question of essential importance for both fundamental studies of relaxor ferroelectrics and their applications to electro-optic devices. The frequency dependence of dielectric response to the bias electric field opens a brand new window for the study of this problem. A novel model from mesoscopic to macroscopic, revealing the relationship between the dielectric permittivity to the applied electric field, temperature, and PNRs, was established based on mean field approximation and the theory of continuum percolation, and not only validates the field-induced percolation and the relaxation time divergency at the freezing temperature, but also predicts the frequency dependence of dielectric response. Unexpectedly, the model reveals the field-enhanced correlation length results in the nonmonotonic behavior of dielectric response, and implies that the increased orientation consistency of dipolar clusters and coercive fields originated from inherent inhomogeneity slow down the relaxation time of PNR reorientation. Considering the multi-scale heterogeneity of PNRs in relaxor, we found that the increased heterogeneity degree reduces the dielectric permittivity, but changes the slope of dielectric response to the bias electric field.

## 1. Introduction

Dielectric properties of relaxor ferroelectrics have been widely studied theoretically and experimentally, owing to their potential for wide application. KTa_1-x_Nb_x_O_3_ (KTN) crystal is a composition-disordered relaxor ferroelectric with perovskite structure. It has attracted considerable attention due to its potential applications in functional devices, including optical switches [1,2,3], electro-optic (EO) modulators [4,5], and deflectors [6,7,8]. All of them benefit from dielectric permittivity that diverges at the Curie temperature. The giant dielectric permittivity is generally believed to originate from the reorientation dynamics of polar nanoregions (PNRs) under the electric field [9]. Below the Burn temperature (T_B_) [10], the PNR begins to rapidly form through the interaction among adjacent dipoles and orients between the states with the same energy, and contributes less to the dielectric permittivity because of violent thermal fluctuation. Dropping to the intermediate temperature (T*) [11], enhanced interaction among the dipole clusters increases the correlation length, giving the PNR local field properties. The PNR will be reoriented under the effect of the electric field, which greatly changes the dielectric permittivity of the KTN crystal, characterized by the obvious deviation from Curie–Weiss law.

The dielectric response to the electric field has received extensive attention owing to the anomalous behavior of paraelectric KTN near the Curie temperature [12,13,14,15,16], and it is closely related to the reorientation dynamics of PNRs. The dielectric responses to the previously reported bias electric field (DRBEF) were completely different: field-induced attenuation [17,18] and field-induced enhancement [8,16]. In an earlier paper, we carried out a more comprehensive investigation of DRBEF and found that it was closely related to the frequency [19]. So far, the micro-mechanism deep insight into the frequency dependence of DRBEF remains unclear.

The reorientation of PNR driven by the electric field should be taken into careful consideration. The reorientation of PNRs can be characterized by relaxation time (τ). Several empirical models, linking the relaxation time to the applied electric field, have been obtained in the study of domain motion [11,20,21,22,23], but failed to clarify the effect of the microscopic mechanism of PNRs on relaxation time. Vogel–Fulcher (V-F) relation describes the relaxation time to the temperature, successfully explaining why the characteristic relaxation time diverges at the freezing temperature, but lacks the electric field effect [24]. Vopsaroiu proposed a general model based on the Pauli master equation, successfully predicting the relationship between the relaxation time and the temperature, the applied electric field, and the critical volume, and proclaimed that the relaxation time decreases monotonously with the electric field [25]. The Debye relaxation equation clearly states that the monotonic decay of relaxation time can only describe the field-enhanced effect in a dielectric response. Moreover, the model cannot explain the divergence of PNR relaxation time at freezing temperature for relaxor. The latest research shows that bias electric field can induce a cubic–tetragonal phase transition for KTN near the Curie temperature, which is similar to the temperature effect [16]. The volume of PNRs increases with the decreased temperature, indicating that the applied bias electric field should have an enlarged effect on PNRs. Therefore, the field-enhanced correlation length of PNRs should be carefully treated.

In this paper, we derive a generalized model of PNR relaxation based on the Pauli master equation and the theory of continuum percolation, successfully predicting the frequency dependence of DRBEF, validating V-F relation and field-induced percolation, and revealing that the field-enhanced correlation length of PNRs plays a vital role in the frequency dependence of the dielectric response to the electric field. 

## 2. Model

Considering PNRs in paraelectric KTN with tetragonal symmetry, it is assumed that PNR can occupy any one of the six states characterized by their easy [100]-type polarization direction. When a nonequilibrium system experiences different possible states, the time evolution of the occupancy probability between the two states can be described by the generalized Pauli master equation [25,26,27,28],

(1) $$\frac{dP_{i}}{dt}=\sum_{i\neq j}[a_{i,j}P_{j}\left(t\right)-a_{i,j}P_{i}\left(t\right)]$$

where 1≤i,j≤k, i and j take integers, k is the number of occupied states of the system, and k=6 in KTN system. $P_{i}\left(t\right)$ and $P_{j}\left(t\right)$ are the probabilities that the system is in state i or j at time t, respectively. $a_{i,j}$ and $a_{j,i}$ are the transition rates from state i to state j and vice versa, respectively. Since reorientation of PNRs is a thermally activated process, $a_{i,j}$ can be derived from Boltzmann theory, as follows:

(2) $$a_{i,j}=v^{0}\exp\left[W_{B}/k_{b}T\right]$$

where $v^{0}=10^{-12}s$. According to Neel’s proposal, W_B_ can be expressed as the product of the PNR volume, polarization, and coercive field [29,30] $W_{B}=E_{c}P_{s}V$. Under the external applied electric field, the relaxation time for reorientation of PNR can be derived from Equation (1),

(3) $$\tau =v_{0}^{-1}\exp[P_{s}\left(E_{c}-E_{app}\right)V/k_{b}T]$$

According to the theory of continuum percolation, the volume should be the function of temperature: $V=V_{0}/\left(1-T_{0}/T\right)$, where $T_{0}$ is the freezing temperature. Then, the relaxation time can be rewritten,

(4) $$\tau =v_{0}^{-1}\exp[\frac{P_{s}\left(E_{c}-E_{app}\right)V}{k_{b}\left(T-T_{0}\right)}]$$

**Figure 1:**
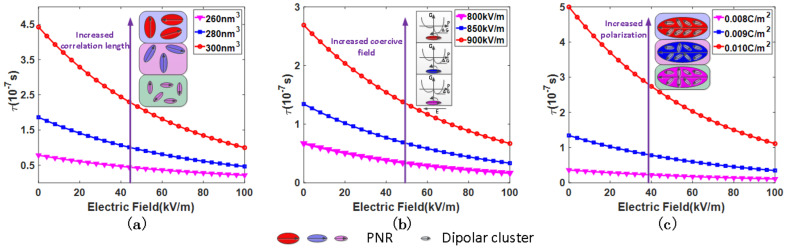
Electric field dependence of relaxation time (**a**) under a different volume of PNRs and (**b**) under a different coercive field. The inset shows the Gibbs free energy versus the polarization, and PNR orients to the direction with lower energy. (**c**) The PNRs overcome the coercive field and reorientate along the direction of electric field under different polarization. The orientation consistency of dipole clusters indicates the polarization. The purple arrow shows the diversity of relaxation time when the same parameter is taken with different values under the same bias electric field.

At $T_{0}$, the relaxation time diverges, indicating the freezing of PNR reorientation. Assuming that the volume of PNR does not change with the electric field, the relationship between the relaxation time of PNR and the electric field is shown in Figure 1. As the electric field increases, the relaxation times of PNR all present a monotonic downward trend, indicating that the applied electric field can activate the reorientation of PNRs. The increased correlation length among the dipolar clusters results in large PNRs, slowing down the relaxation of PNRs, as shown in Figure 1a. The coercive field originates from the inherent inhomogeneity, resulting in the asymmetry of the potential energy surface and the orientation of PNRs towards the direction with lower energy, as shown in the inset of Figure 1b. The increased coercive field hinders the reorientation of PNRs. The large polarization implies that the elementary dipolar clusters in PNR tend to orient one direction. Figure 1c illustrates that alignment consistency of the elementary dipolar clusters which makes the reorientation of PNRs more difficult.

**Figure 2:**
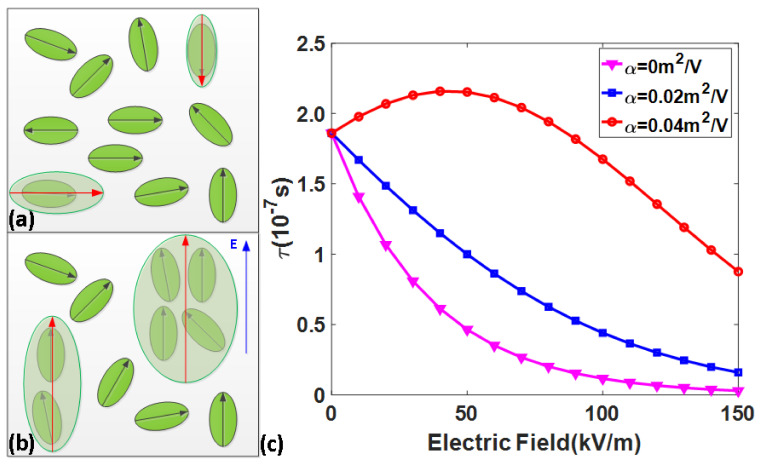
(**a**) Small PNRs without electric field. (**b**) Enlarged PNRs under the electric field. (**c**) Electric field dependence of relaxation time under different field-enlarged rates of volume. The black and red arrows indicate the polarization direction of dipoles and PNRs, and the blue arrow indicates the electric field direction.

According to the Debye relaxation model, Equation (4) reveals that the bias electric field can only increase the dielectric permittivity, that is, the field-enhanced effect. This shows that the relaxation time model is not perfect. From the macroscopic point of view, the bias electric field can induce the cubic to tetragonal phase transition near the Curie temperature, and the effect of the electric field is similar to the cooling effect [16]. From the microscopic point of view, the field-induced percolation theory predicts that the applied electric field can rearrange the random-oriented PNR from disordered to ordered state, enhance the correlation interaction between PNRs, and make them condense into large PNR [31]. Figure 2a illustrates that the volume of PNR is small when the external electric field is zero, and the applied electric field enlarges the PNR, as shown in Figure 2b. Therefore, the relaxation time of PNR must introduce the enlarged effect of bias electric field on the volume. Equation (4) can be further amended by assuming a linear relationship between the volume and the field.

(5) $$\tau =v_{0}^{-1}e^{[(E_{c}P_{s}-P_{s}Eapp)/k_{b}\left(T-T_{0}\right)]\left(V_{0}+\alpha E\right)}$$

where α is the field-enlarged rate of PNR volume. It is assumed that the relationship between the volume of PNR and electric field is $V=V_{0}+\alpha *10^{-29}*E_{app}$. Setting α to be 0, 0.02, and 0.04, respectively, the electric field dependence of relaxation time is illustrated in Figure 2. The previous analysis indicates that the increased electric field and volume have opposite effects on the PNR reorientation. Figure 2c shows that the activation of the electric field plays a leading role: when the field-enlarged rate is low, the relaxation time decreases monotonically. However, when the field-enlarged effect rate is high, the increased volume slows down the PNR reorientation in the low electric field range. However, in the high electric field range, the effect of field-activated PNR reorientation exceeds the effect of increased volume hindering PNR reorientation, and the relaxation time decays gradually.

According to the Debye model, the dielectric permittivity can be expressed as follows,

(6) $$\varepsilon^{\prime }=\varepsilon_{\infty }+\frac{\varepsilon_{s}-\varepsilon_{\infty }}{1+\omega^{2}\tau^{2}}$$

where $\varepsilon_{\infty }$ is the optical frequency dielectric permittivity, and $\varepsilon_{s}$ is the static dielectric permittivity contributed by PNR, which can be expressed as: $\varepsilon_{s}=P_{s}^{2}V/k_{b}\left(T-T_{0}\right)$. Dielectric permittivity is negatively correlated with the relaxation time (τ). From this point of view, the electric field enhances the correlation between PNRs. Large PNR then forms, resulting in a longer relaxation time, as seen in Figure 1a. With the increment of electric field, the relaxation time first increases and then decays, and the dielectric permittivity contributed by PNR reorientation first decreases and then increases. The competition mechanism of increasing the electric field and volume for PNR reorientation may explain the frequency dependence of the dielectric response to the bias electric field.

## 3. Experimental Results and Discussion

**Figure 3:**
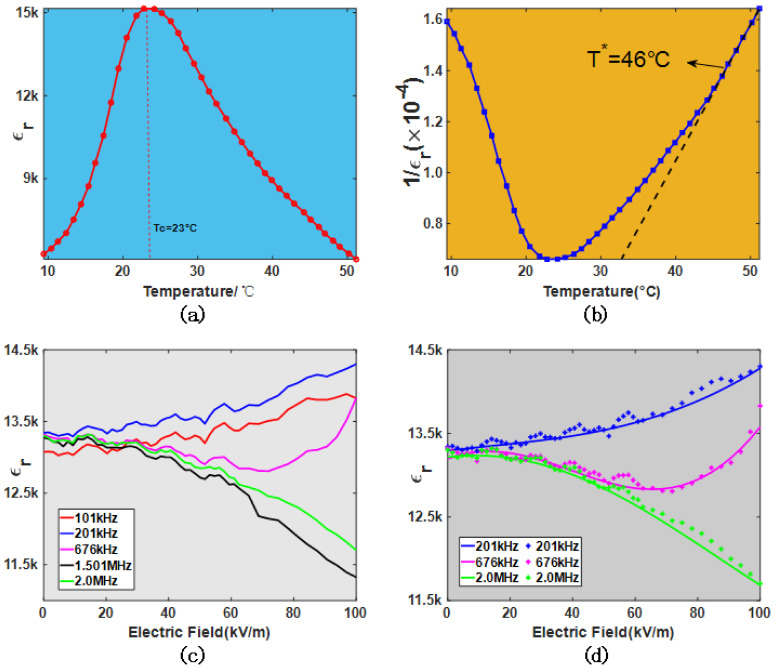
The temperature dependence of the dielectric permittivity (**a**) $\varepsilon_{r}$ and impermeability (**b**) $1/\varepsilon_{r}$. (**c**) The frequency dependence of the dielectric response to the bias electric field. The red dotted line indicates the Curie temperature, the black dotted line represents the Curie-Weiss law, and the black arrow indicates the intermediate temperature. (**d**) Fitting result at 201 kHz, 676 kHz, and 2 MHz. The plus sign indicates the experimental data, and the solid line represents the result fitted by Equation (6).

A high-quality KTN:Cu was grown by the top-seeded solution growth (TSSG) method. The KTN crystal was cut into a tetragon with dimensions of 7^(x)^ mm × 4.7^(y)^ mm × 3.2^(z)^ mm along the crystallographic directions <100>. The 7^(x)^ mm × 4.7^(y)^ mm surfaces were coated by a silver electrode, and other surfaces were optically polished. The dielectric permittivity spectrum versus temperature was measured by an LCR meter (TH2839) with a small AC signal of 1V and 1kHz, as shown in Figure 3a. The corresponding position of the dielectric peak, marked by a dotted line, is the Curie temperature, $T_{c}=23\;{}^{\circ}C$. Further, Figure 3b illustrates the $1/\varepsilon_{r}$ versus temperature, $T^{*}=46\;{}^{\circ}C$, which was defined as the point at which $\varepsilon_{r}$ deviated from the Curie–Weiss law, implying that the reorientation dynamics of PNR are affected by the electric field. Figure 3c shows the frequency dependence of the dielectric response to the bias electric field under 26 °C. At 101 kHz and 201 kHz, the dielectric permittivity increases monotonously with the bias electric field. However, at 676 kHz, the nonmonotonic behavior of the dielectric response appears, and the dielectric permittivity first decreases to the minimum and then increases, indicating that there seems to be a competitive mechanism to regulate the dielectric permittivity. As the frequency continues to increase, the dielectric permittivity decreases monotonously, such as 1.501 MHz and 2.0 MHz.

Assuming that all PNRs have the same size $V_{0}$, the experimental data of the dielectric response to the bias electric field at five frequencies were fitted with Equation (6). Figure 3d shows the fitting results at 201 kHz, 676 kHz, and 2.0 MHz. The calculated parameters are listed in Table 1. The quality of the fits to the experimental data is excellent, implying that the proposed model successfully describes the frequency dependence of the dielectric response to the bias electric field.

**Figure 4:**
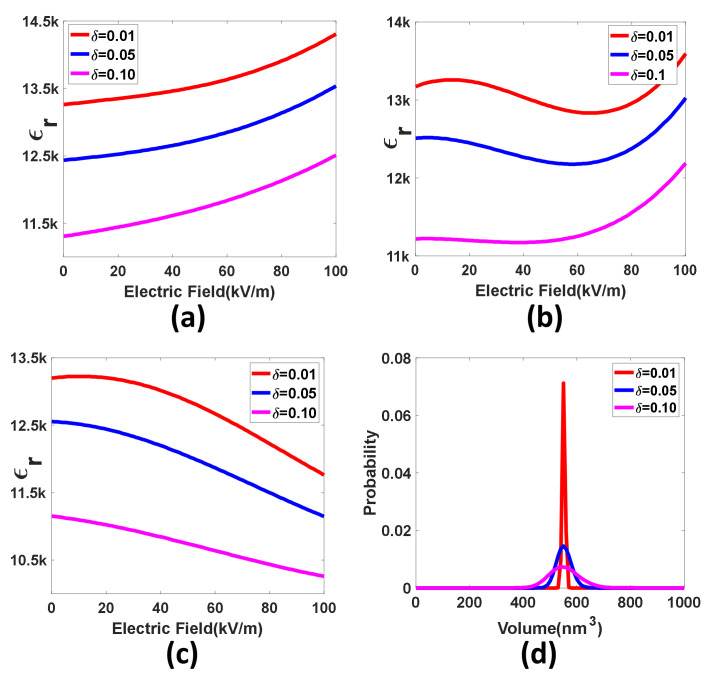
The dielectric response to the bias electric field for a different distribution width: (**a**) 201 kHz, (**b**) 676 kHz, and (**c**) 2 MHz. (**d**) The probability distribution of volume for a different distribution width. We performed the numerical simulations using the parameters calculated in Table 1.

Actually, multi-scale inhomogeneity of PNR in relaxor has been proven [32]. Therefore, the influence of the multi-scale inhomogeneity on the macroscopic dielectric response to the bias electric field should be carefully considered. The dielectric response can be expressed as

(7) $$\varepsilon_{r}=\underset{i}{\sum }p_{i}\left(V\right)\varepsilon^{\prime }$$

where $p_{i}\left(V\right)$ represents the probability of PNR with volume *V*. The distribution of volumes of PNRs can be described by a log Gaussian distribution function [33]. Furthermore, the averaged dielectric permittivity can be expressed as

(8) $$\varepsilon_{r}=\int_{v^{-}}^{v^{+}}f\left(V|\mu ,\sigma \right)\left(\varepsilon_{\infty }+\frac{\varepsilon_{s}-\varepsilon_{\infty }}{1+\omega^{2}\tau^{2}}\right)dx,$$

where μ controls the position of maximum probability, as shown in Figure 4d. Figure 4a,b show the effect of the size distribution width of PNRs on the dielectric response to the bias electric field for different frequencies. Figure 4a suggests that the increasing heterogeneity decreases the dielectric permittivity, but slightly improves the field-enhanced rate. Figure 4b illustrates that the increasing inhomogeneity weakens the nonmonotonic dynamic behavior of the dielectric response to the bias electric field. Figure 4c indicates that the increasing heterogeneity reduces the field-induced attenuation effect, and tends to make the dielectric response to the bias electric field unanimous. To obtain large permittivity, the size distribution of PNRs should be as narrow as possible, but the bias electric field can significantly change the permittivity, which is not conducive to decreasing the slope of the dielectric response to the electric field. 

The simulation and analysis are carried out on the premise that all characteristic parameters of PNR of any volume are consistent, such as $\varepsilon_{\infty },\;P_{m},\;E_{c},\;T_{0},\;\alpha$. The real value of the characteristic parameters of PNRs may change with the volume. Therefore, the numerical results are not absolute in this section and they are intended only for guidance, and these findings are very important as they suggest the distribution width dependence of the dielectric response to the bias electric field, which should be seriously considered in the design, operation, and stability consideration of dielectric tunable devices, electro-optic modulators, and deflectors based on relaxor ferroelectrics. In addition, the dielectric model developed here is based on a Debye relaxation. However, the relaxation is more complex than Debye relaxation in most systems. Nevertheless, the calculated results based on the Debye model are useful to understand the effect of multi-scale heterogeneity on the dielectric permittivity.

## 4. Conclusions

To conclude, the proposed microscopic model comprehensively describes the relationship between dielectric response to the electric field, temperature, and PNR relaxation, and validates the percolation theory and relaxation divergency at the freezing temperature. Furthermore, the model successfully describes the frequency dependence of the dielectric response to the bias electric field. The field-enlarged effect of PNR volume can result in nonmonotonic behavior in the dielectric response. Furthermore, the inhomogeneity of PNRs reduces the dielectric permittivity and changes the slope of the dielectric response to the bias electric field. The findings provide a better understanding of the field-driven dynamics of PNRs which change the dielectric properties of relaxors, and has guiding significance for designing nanodomain structure to control the dielectric behavior.

## Figures and Tables

**Table 1:** The calculated parameters by fitting Equation (6).

Parameters	$\varepsilon_{\infty }$	$E_{c}\left(kV/m\right)$	$P_{S}\left(C/m^{2}\right)$	$V_{0}\left(nm^{3}\right)$	$T_{0}\left(K\right)$	$\alpha \left(m^{2}/V\right)$
101 kHz	4998	900.3	0.007707	361	288.2	0.0503
201 kHz	4997	722.2	0.006525	519.1	287.9	0.09605
676 kHz	3000	454.4	0.004374	606.5	293.3	0.2234
1.501 MHz	2200	996.3	0.01025	240.2	285.4	0.04852
2 MHz	2145	873.5	0.009247	276	286.5	0.05921

## Data Availability

Data are contained within the article.
